# The Effect of Gender on Mesenchymal Stem Cell (MSC) Efficacy in Neonatal Hyperoxia-Induced Lung Injury

**DOI:** 10.1371/journal.pone.0164269

**Published:** 2016-10-06

**Authors:** Ibrahim Sammour, Santhosh Somashekar, Jian Huang, Sunil Batlahally, Matthew Breton, Krystalenia Valasaki, Aisha Khan, Shu Wu, Karen C. Young

**Affiliations:** 1 Department of Pediatrics, University of Miami Miller School of Medicine, Miami, FL, United States of America; 2 Batchelor Children’s Research Institute, University of Miami Miller School of Medicine, Miami, FL, United States of America; 3 The Interdisciplinary Stem Cell Institute, University of Miami Miller School of Medicine, Miami, FL, United States of America; Medical University Innsbruck, AUSTRIA

## Abstract

**Background:**

Mesenchymal stem cells (MSC) improve alveolar and vascular structures in experimental models of bronchopulmonary dysplasia (BPD). Female MSC secrete more anti-inflammatory and pro-angiogenic factors as compared to male MSC. Whether the therapeutic efficacy of MSC in attenuating lung injury in an experimental model of BPD is influenced by the sex of the donor MSC or recipient is unknown. Here we tested the hypothesis that female MSC would have greater lung regenerative properties than male MSC in experimental BPD and this benefit would be more evident in males.

**Objective:**

To determine whether intra-tracheal (IT) administration of female MSC to neonatal rats with experimental BPD has more beneficial reparative effects as compared to IT male MSC.

**Methods:**

Newborn Sprague-Dawley rats exposed to normoxia (RA) or hyperoxia (85% O2) from postnatal day (P) 2- P21 were randomly assigned to receive male or female IT bone marrow (BM)-derived green fluorescent protein (GFP^+^) MSC (1 x 10^6^ cells/50 μl), or Placebo on P7. Pulmonary hypertension (PH), vascular remodeling, alveolarization, and angiogenesis were assessed at P21. PH was determined by measuring right ventricular systolic pressure (RVSP) and pulmonary vascular remodeling was evaluated by quantifying the percentage of muscularized peripheral pulmonary vessels. Alveolarization was evaluated by measuring mean linear intercept (MLI) and radial alveolar count (RAC). Angiogenesis was determined by measuring vascular density. Data are expressed as mean ± SD, and analyzed by ANOVA.

**Results:**

There were no significant differences in the RA groups. Exposure to hyperoxia resulted in a decrease in vascular density and RAC, with a significant increase in MLI, RVSP, and the percentage of partially and fully muscularized pulmonary arterioles. Administration of both male and female MSC significantly improved vascular density, alveolarization, RVSP, percent of muscularized vessels and alveolarization. Interestingly, the improvement in PH and vascular remodeling was more robust in the hyperoxic rodents who received MSC from female donors. In keeping with our hypothesis, male animals receiving female MSC, had a greater improvement in vascular remodeling. This was accompanied by a more significant decrease in lung pro-inflammatory markers and a larger increase in anti-inflammatory and pro-angiogenic markers in male rodents that received female MSC. There were no significant differences in MSC engraftment among groups.

**Conclusions:**

Female BM-derived MSC have greater therapeutic efficacy than male MSC in reducing neonatal hyperoxia-induced lung inflammation and vascular remodeling. Furthermore, the beneficial effects of female MSC were more pronounced in male animals. Together, these findings suggest that female MSC maybe the most potent BM-derived MSC population for lung repair in severe BPD complicated by PH.

## Introduction

Bronchopulmonary dysplasia (BPD) was first described in infants ventilated for hyaline membrane disease (HMD) by Northway and colleagues in 1967 [1]. This disease is a chronic multifactorial disorder that affects 12–32% of infants less than 32 weeks of gestation, with most cases occurring in extremely low birth weight infants [2]. Over the last 2 decades, the characteristics of BPD have been modified by the use of prenatal corticosteroids, postnatal surfactant and gentle ventilation strategies [3, 4]. This “new” BPD is characterized by alveolar simplification, increased alveolar diameter and abnormal vascular development [5]. Extremely low birth weight (ELBW) infants are now surviving in greater numbers and BPD with its associated pulmonary arterial hypertension (PH) now constitute a major source of morbidity and late mortality [3, 6]. Unfortunately, therapeutic options are limited, with survivors experiencing an increased risk of adverse neurodevelopmental outcomes [7].

Gender variability in BPD incidence has been described for several decades. Animal studies have demonstrated that gender not only influences lung maturation but also susceptibility to lung diseases [8, 9]. Male mice are more susceptible to hyperoxic lung injury than female mice [10]. Population based data from the Australian and New Zealand Neonatal Network show that preterm males are more likely than females to develop BPD [11]. Similarly, in the MOSAIC cohort from ten European regions, multivariate analysis reveal that male gender is associated with an increased incidence of BPD [12]. Interestingly, although the exact mechanisms underlying the gender-specific differences in BPD incidence remain unknown, female mice express less pro-inflammatory and oxidative stress markers in response to hyperoxia [10]. Whether the efficacy of BPD therapies is modified by their interaction with the gender of the host is unclear.

Mesenchymal stem cells (MSC) have been studied extensively as therapeutic mediators in multiple diseases [13–15]. These cells are particularly attractive for therapy as they are relatively easy to expand, possess potent anti-inflammatory, immunomodulatory, and pro-angiogenic effects, with low risk of inciting the immune system [16, 17]. In rodent models of BPD, early administration of MSC is associated with improved alveolarization, decreased vascular remodeling, promotion of angiogenesis and improvement of PH [18–20]. In a recent phase-1 clinical trial, administration of umbilical cord derived MSC to preterm infants at risk for BPD was shown to be safe and feasible, with some potential beneficial effects [21]. Data however remains limited on the most efficacious MSC population for lung regeneration in BPD.

Gender-specific differences in stem cell expansion capacity, differentiation potential, and secretome have been reported in the literature [22, 23]. This sex-related difference in stem cell function is influenced by the stem cell population, recipient responses and the disease process. Female muscle derived stem cells regenerate skeletal muscle more efficiently than male cells but transplantation of male stem cells into female recipients or pre-treatment of male cells with estradiol fail to yield comparable regeneration, implying that gender-related differences in stem cell function may not be entirely dependent on sex hormones [24]. In a mouse model of myocardial infarction, infusion of female derived MSC was associated with more pronounced improvement in left ventricular dysfunction [25]. Similarly, in a rat model of endotoxin mediated cardiac dysfunction, female MSC showed greater cardiac protection against endotoxemic injury and this was accompanied by an improvement in the myocardial anti-apoptotic profile [26]. In vitro data also shows that female MSC exposed to either lipopolysaccharide or hypoxia have less apoptosis, release less tumor necrosis factor alpha (TNF-α) and more vascular endothelial growth factor (VEGF) than male MSC [27]. Whether the therapeutic efficacy of MSC in attenuating lung injury in an experimental model of BPD is influenced by the sex of the donor or recipient is unknown.

In this study, we hypothesized that female BM-derived MSC would have greater lung regenerative effects than male BM-derived MSC in a model of BPD, and the effects would be preferential to male recipients. We demonstrate in vitro that female MSC secrete more anti-inflammatory and pro-angiogenic factors as compared to male MSC. In vivo, we also show that female MSC have greater anti-inflammatory and pro-angiogenic effects as compared to male MSC in neonatal pups with experimental BPD. Moreover, female MSC more robustly improves neonatal hyperoxia-induced PH and vascular remodeling. Interestingly, in keeping with our hypothesis, female MSC improved vascular remodeling to a greater degree in male recipients than male MSC. These findings have important clinical implications for cell-based therapies in preterm infants with BPD.

## Methods

### Animals

Adult male and female Green Fluorescent Protein (GFP) transgenic and pregnant female Sprague Dawley rats were purchased from The Rat Resource and Research Center (Columbia, MO). Animals were treated according to National Institutes of Health guidelines. The protocol was approved by the Animal Care and Use Committee (ACUC) of the University of Miami Miller School of Medicine.

### Experimental Procedure

Randomly chosen Sprague-Dawley pups from 16 litters (N = 142) were assigned at birth to Room air (RA) or hyperoxia (85–90% O2) from P2 to P21. Pups were housed in a plexiglass chamber with O2 monitoring. Litters for each experimental group were limited to 10 pups to control for the effect of litter size on nutrition and growth. Studies were repeated with several litters in order to obtain an equivalent number of pups per experimental condition. Dams were rotated every 48 hours to standardize the nutrition provided to each litter. Oxygen exposure was continuous with brief intermittent interruptions for animal care (<10 min/day). After 3 weeks in the designated exposure, the litters were removed and studied for hemodynamic measurements and morphometry. The sex of the pups was determined by examining external and internal genitalia.

### MSC Administration

MSC obtained from the bone marrow of adult (6 to 8 week old) male and female GFP^pos^ Sprague-Dawley rats were isolated and cultured as previously described [28]. For intra-tracheal (IT) injection, fourth passage cells were thawed, assessed for viability, and washed with phosphate buffered saline (PBS). They were then re-suspended in PBS at 1 X10^6^ viable cells/50 μl. A single IT dose of 50 μl was utilized for administration.

On P7, the pups were anesthetized with ketamine and xylazine via intraperitoneal injection. The trachea was exposed through a small midline incision on the neck. MSC (1 X10^6^ cells/50 μl), or phosphate buffered saline/PL (50 μl) was delivered by tracheal puncture with a 30-gauge needle. Pups were placed in a warmed plastic chamber under normoxic or hyperoxic conditions for recovery. Once the pups were fully awake, they were returned to their dams.

### Hemodynamic Measurements

Right ventricular systolic pressure (RVSP) was evaluated as previously described [29]. Briefly, a thoracotomy was performed and a 22-gauge needle connected to a pressure transducer was inserted into the right ventricle. RVSP was measured and recorded on a Gould polygraph (model TA-400; Gould instruments, Cleveland, OH).

### Morphometric Analysis

Lung morphometry was performed as previously described [30]. Briefly, lungs were inflated and perfused with 4% paraformaldehyde (PFA) at a pressure of 15 cmH_2_O for five min. The samples were allowed to sit in PFA for 24 hours prior to serial dehydration in ethanol solutions the following day. The lungs were then embedded in paraffin.

Serial 5 μm-thick paraffin-embedded sections obtained from the lung were stained with hematoxylin and eosin. Images from 10 randomly selected, non-overlapping parenchymal fields were acquired from two lung sections of each animal at 20x magnification. Care was taken to exclude major bronchioles, vessel and artifacts from the field. Images were captured by a blinded observer, and the mean linear intercept, (MLI), a measure of inter-alveolar wall distance, and the radial-alveolar count (RAC), a measure of alveolarization were analyzed.

### Pulmonary Angiogenesis

Vascular density was evaluated as previously described [19]. Briefly, lung sections were stained with polyclonal rabbit antihuman Von Willebrand Factor (VWF; Dako, Carpinteria, CA), a marker of endothelial cells, and 4’6-di-amidino-2-phenylindole (DAPI;Vector Laboratories), a marker of cell nuclei. Five randomly selected non-overlapping parenchymal fields were evaluated from lung sections of each animal. The number of blood vessels (20–50 μm in diameter) in each high-power field was counted by a blinded observer as previously described [31].

### Pulmonary Vascular Remodeling

Pulmonary vascular remodeling was evaluated as previously described [19]. Briefly, lung sections were stained with polyclonal rabbit antihuman VWF (Dako), mouse anti α-Smooth Muscle Actin (SMA) (Sigma, St Louis, MO), a marker of smooth muscle in the medial wall of vessels, and DAPI (Vector Laboratories). Five randomly selected non-overlapping parenchymal fields were evaluated from lung sections of each animal. The blood vessels (20–50 μm in diameter) in each high-power field were counted by a blinded observer and the degree of muscularization assessed at that time [31]. Medial wall thickness was measured in 20 randomly selected arterioles (20–50 μm in diameter) at 40X magnification as previously described [32]. Briefly, the thickness of the medial layer of the arteriole was identified using SMA fluorescence and measured at its thickest portion. The average diameter of the arteriole was also obtained. The medial wall thickness index was calculated as 2 X measured thickness of the medial layer / average diameter of the vessel X 100%.

### ELISA & Western Blot

Bone marrow derived MSC from male and female donors (N = 6) were cultured in fetal bovine serum (FBS) for 24 hours under normoxic-conditions. The cells were trypsinized and protein isolated as previously described [19]. The protein concentration of VEGF and Interleukin-10 (IL-10) in male and female MSC as well as homogenized lungs was determined by Enzyme Linked Immunosorbent Assay (ELISA). VEGF and IL-10 ELISA kits were obtained from Abcam (Cambridge, MA). The protein expression of Interleukin-1β (IL-1β) in lung homogenates was determined by Western Blot using a mouse monoclonal antibody obtained from Cell Signaling (1:1000, Beverley, MA), with β-Actin acting as a normalization protein (Sigma-Aldrich, St. Louis, MO). The intensities of protein bands were quantified by densitometry using Quantity One Imaging Analysis Program (Bio-Rad, Hercules, CA).

### Cell Engraftment

Serial five micrometer (μm) paraffin-embedded sections obtained from the upper and lower lobes of the lungs were dewaxed and rehydrated in descending grades of alcohol. Following antigen retrieval and blocking of non-specific binding sites with a protein blocker, the lung sections were stained anti-GFP antibody (1:50; Santa Cruz Biotechnology, Santa Cruz, CA). Engrafted GFP^pos^ cells were imaged with a confocal microscope (Leica DMI 6000, Mannheim, Germany) and 5 random fields per section were counted by a blinded observer and expressed as a percentage of all nuclei present in that field.

### Statistical Analysis

Data are expressed as mean ± standard deviation and were analyzed by two-way ANOVA with *post hoc* analysis (Holm-sidak). *P* values (*P)* ≤0.05 were considered statistically significant. Statistical analysis was performed using SigmaStat software (SyStat Software, San Jose, CA).

## Results

### Female MSC express more VEGF and IL-10

**Fig 1:**
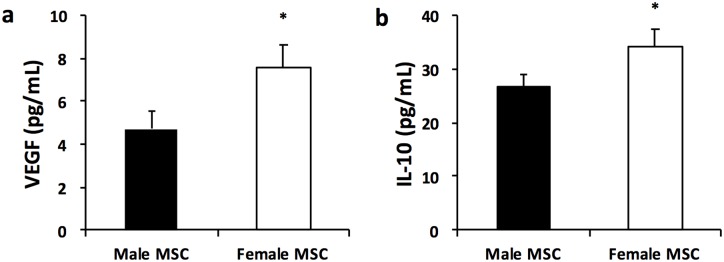
Female MSC produce more VEGF and IL-10 after 24 hours in culture. (a) VEGF concentration as measured by ELISA in male and female MSC (b) IL-10 concentration as measured by ELISA in male and female MSC (* *P* < 0.05; Male vs Female MSC, N = 3 experiments/group).

Female MSC grown for 24 hours under normoxic conditions had higher VEGF concentration than male MSC (7.6 ± 0.8 vs 4.7 ± 1.0 pg/mL, female vs male MSC; *P* < 0.05, N = 3/group), Fig 1a. Female MSC also secreted more of the anti-inflammatory cytokine, IL-10 than male MSC (34.2 ± 3.0 vs 26.8 ± 2.1 pg/mL, female vs male MSC; *P* < 0.05, N = 3/group), Fig 1b.

These findings suggest that female MSC possess greater pro-angiogenic and anti-inflammatory effects than their male derived counterparts.

### Female and male MSC similarly improve alveolarization

**Fig 2:**
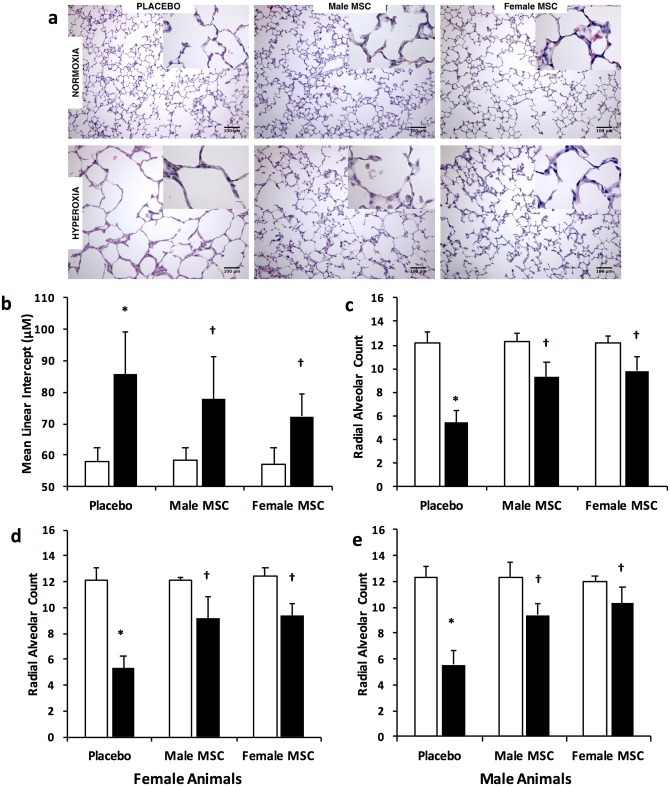
Intra-tracheal (IT) administration of male or female MSC similarly improves alveolarization. (a) Hematoxylin and eosin stained lung sections demonstrating improved alveolar structure in hyperoxia-exposed pups treated with IT male or female MSC. Original magnification X 200. Bars = 100 μm. IT male or female MSC similarly increased mean linear intercept (b) and decreased radial alveolar count (c). IT male or female MSC similarly improve RAC in female (d) and male (e) animals. (*P < 0.05; Room air (RA) vs hyperoxia- placebo (PL), † *P* <0.05; hyperoxia -PL vs hyperoxia male or female MSC). White bars are RA and black bars are hyperoxia.

Hyperoxia-exposed placebo treated rats had marked alveolar simplification (Fig 2a). This was evidenced by an increase in MLI (58 ± 4 vs 86 ± 14 μm, RA vs hyperoxia-PL; *P* < 0.05, N = 10/group), and a decrease in RAC (12.2 ± 0.9 vs 5.4 ± 1.0, RA vs hyperoxia-PL; *P* < 0.05, N = 10/group), Fig 2b and 2c. Administration of male or female MSC to hyperoxic pups reduced MLI (77.9 ± 13.6 vs 72.4 ± 7.2 μm, hyperoxia male MSC vs hyperoxia female MSC; N = 10/group) and increased RAC to similar degree (9.3 ± 1.3 vs 9.8 ± 1.2, hyperoxia male MSC vs hyperoxia female MSC; N = 10/group), Fig 2b and 2c. This suggests that female derived MSC are as effective as male derived MSC in promoting alveolarization in neonatal hyperoxia-induced lung injury. Moreover, subgroup analysis performed to study interactions between recipient and donor sexes revealed no significant differences in the alveolarization noted between male and female MSC when administered to hyperoxia exposed female or male pups (N = 5/group), Fig 2d and 2e respectively.

### Female and male MSC similarly improve angiogenesis

**Fig 3:**
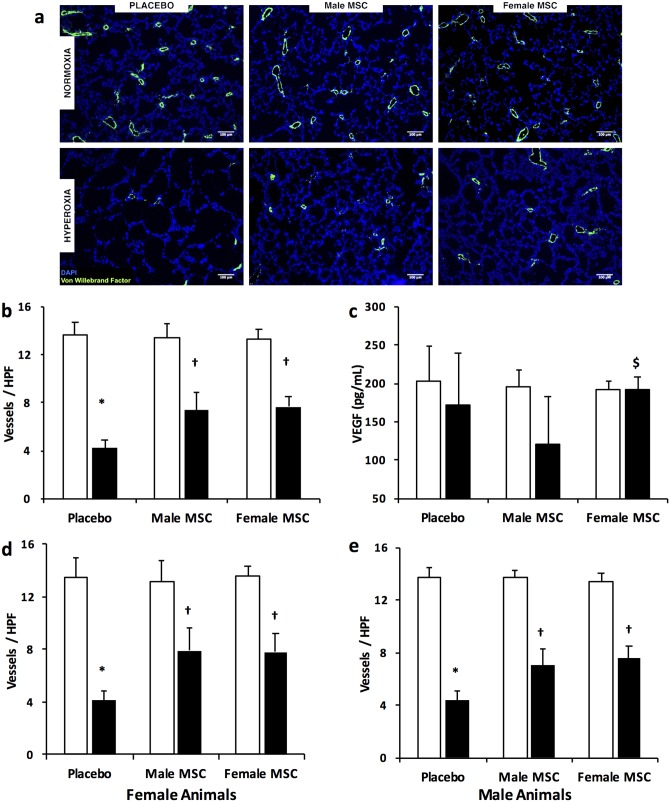
IT male or female MSC similarly improve lung angiogenesis. (a) Lung sections stained with Von Willebrand Factor (green) and 4’6-diamidino-2-phenylindole (DAPI: blue), demonstrating improved vascular density in hyperoxia-exposed pups treated with male and female MSC at P7. Original magnification X 100. Bars = 100 μm. (b) IT male and female MSC similarly increased lung vascular density in hyperoxic pups. (c) Female MSC normalizes lung VEGF concentration in hyperoxia. Female and male MSC exhibit similar efficacy in improving lung angiogenesis in female (d) and male (e) animals. (*P < 0.05: RA vs hyperoxia-PL; † *P* <0.05: hyperoxia-PL vs hyperoxia male or female MSC; $ *P* <0.05: hyperoxia male MSC vs hyperoxia female MSC). White bars are RA and black bars are hyperoxia.

Exposure of placebo treated rats to hyperoxia caused vascular pruning (Fig 3a) as demonstrated by a reduction in vascular density (13.2 ± 1.1 vs 4.2 ± 0.7 vessels per HPF, RA vs hyperoxia-PL; *P* <0.05, N = 10/group), Fig 3b. Treatment with male or female MSC improved angiogenesis to a similar degree. (7.4 ± 1.5 vs 7.7 ± 0.8 vessels per HPF, hyperoxia male MSC vs hyperoxia female MSC; N = 10/group). There was no difference in VEGF concentration between RA and hyperoxia-exposed placebo treated animals (202 ± 46 vs 172 ± 67 pg/mL, RA vs hyperoxia -PL, N = 5-6/group), Fig 3c. Administration of female MSC significantly increased lung VEGF concentration (120 ± 62 vs 193 ± 15 pg/mL, hyperoxia male MSC vs hyperoxia female MSC; *P* < 0.05, N = 7-9/group) Fig 3c. Interestingly, although hyperoxia exposed male animals treated with female MSC had higher pulmonary VEGF levels (117 ± 71 vs 198 ± 12, pg/mL; hyperoxia male MSC vs hyperoxia female MSC; *P* < 0.05, N = 4-5/group), and there were trends to higher pulmonary VEGF levels in hyperoxia exposed female pups treated with female MSC (125 ± 64 vs 186 ± 19 pg/mL; hyperoxia male MSC vs hyperoxia female MSC; N = 4/group), the improvement in angiogenesis was not affected by the sex of the recipient, Fig 3d and 3e.

### Female MSC are superior to male MSC in attenuating pulmonary hypertension

**Fig 4:**
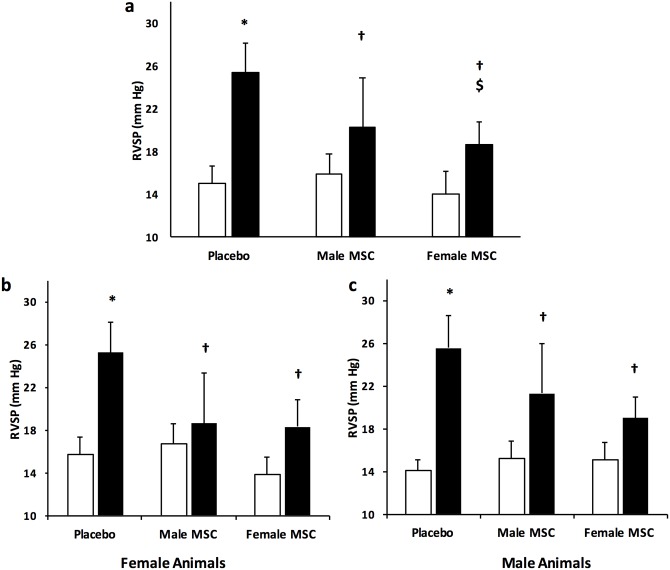
Female MSC have more marked effects on pulmonary hypertension. (a) Female MSC improve right ventricular systolic pressure (RVSP) to a greater degree than male MSC. The efficacy of MSC in improving RVSP in female (b) and male (c) animals is not dependent on the sex of the recipient. (*P < 0.05: RA vs hyperoxia-PL; † *P* <0.05: hyperoxia-PL vs hyperoxia male or female MSC; $ *P* <0.05: hyperoxia male MSC vs hyperoxia female MSC). White bars are RA and black bars are hyperoxia.

PH is a prominent feature of severe lung injury. Right ventricular systolic pressure is a surrogate for pulmonary artery pressure. Exposure of placebo treated animals to hyperoxia was associated with the development of PH (RVSP: 15 ± 2 vs 25 ± 3 mmHg, RA (N = 32) vs hyperoxia-PL (N = 15); *P* < 0.05), Fig 4. Treatment with male or female MSC significantly improved PH. Interestingly, in keeping with our hypothesis, female MSC improved RVSP to a greater degree than male MSC (21 ± 4.0 vs 19 ± 2 mmHg, hyperoxia male MSC vs hyperoxia female MSC; *P* < 0.05, N = 10–11 / group), Fig 4a. This suggests that female MSC may be more beneficial in treating PH complicating BPD.

Subgroup analysis revealed that the improvement in PH with male or female MSC was not dependent on the sex of the recipient animal (N = 5-7/group), Fig 4b and 4c respectively.

### Female MSC are superior to male MSC in improving vascular remodeling

**Fig 5:**
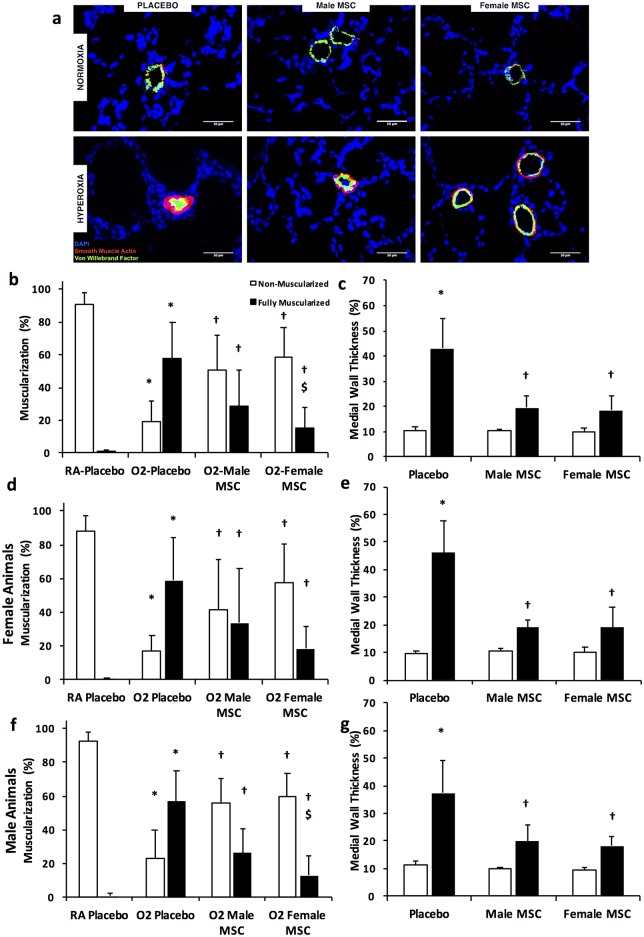
Female MSC exhibit greater anti-remodeling effects. (a) Lung sections stained with Von Willebrand Factor (green), α-smooth muscle actin (red), and DAPI (blue), demonstrating improved vascular remodeling in hyperoxia-exposed pups treated with male or female MSC. Original magnification X400. (b) Female MSC are superior to male MSC in reducing the percentage of fully muscularized blood vessels. (c) IT male or female MSC improved medial wall thickness of vessels (20–50 μm). In female pups, IT male or female MSC similarly improve the percentage of muscularized vessels (d), and the medial wall thickness (e). In male pups, IT female MSC are superior to male MSC in improving the percentage of muscularized blood vessels (f), while being as effective in reducing the medial wall thickness (g). (*P < 0.05: RA vs hyperoxia-PL; † hyperoxia-PL vs hyperoxia male or female MSC; $ *P* <0.05: hyperoxia male MSC vs hyperoxia female MSC). White bars are RA and black bars are hyperoxia unless otherwise specified.

Vascular remodeling is a prominent feature of HILI. Placebo- treated hyperoxic animals had decreased percentage of non-muscularized vessels (90± 7 vs 20 ± 12%, RA vs hyperoxia-PL; *P* < 0.05, N = 10 / group), Fig 5a and 5b. This was accompanied by an increase of fully muscularized blood vessels in the 20–50 μm range (0.7 ± 1 vs 58% ± 22%, RA vs hyperoxia-PL; *P* < 0.05, N = 10 / group), Fig 5a and 5b. Interestingly, although treatment with male or female MSC decreased medial wall thickening (Fig 5c) to a similar degree, the number of fully muscularized blood vessels were markedly less in hyperoxic pups that received female MSC (30 ± 22 vs 16± 12%, hyperoxia male MSC vs hyperoxia female MSC; *P* < 0.05, N = 10 / group), Fig 5b.

Subgroup analysis revealed that although there were trends towards less fully muscularized vessels in hyperoxia exposed female pups receiving female MSC, this was not statistically significant, Fig 5d. The degree of improvement of medial wall thickness with male or female MSC was also not different in female or male recipients, Fig 5e and 5g. In contrast, hyperoxia exposed male pups receiving female MSC had significantly less fully muscularized vessels than male MSC treated animals (26 ± 14 vs 13 ± 11%, hyperoxia male MSC vs hyperoxia female MSC; *P* < 0.05, N = 5 / group), Fig 5f. This suggests that female MSC are superior to male MSC in improving hyperoxia-induced pulmonary vascular remodeling especially in male recipients.

### Female MSC Possess More Anti-inflammatory Properties

**Fig 6:**
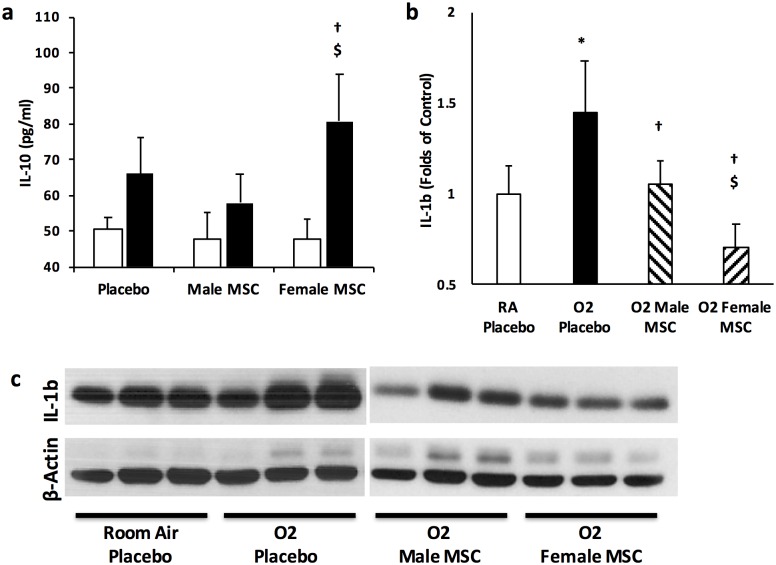
IT MSC and lung inflammation. (a) Markedly greater lung IL-10 concentration in pups treated with female as compared to male MSC. (b) Female MSC decrease lung IL-1β expression to a greater degree than male MSC (c) Representative Western blot demonstrating a more marked decrease in lung IL-1β expression in hyperoxic pups treated with female MSC. β-actin is utilized as the normalization protein. (*P < 0.05: RA vs hyperoxia-PL; † hyperoxia-PL vs hyperoxia male or female MSC; $ *P* <0.05: hyperoxia male MSC vs hyperoxia female MSC; N = 5-6/group). White bars are RA and black bars are hyperoxia.

IL-10 is a pleiotropic regulatory cytokine known to be secreted by BM- derived MSC [33]. It down-regulates the expression of pro-inflammatory cytokines. Since female MSC secreted more IL-10 than male MSC, we next sought to ascertain whether its beneficial anti-inflammatory effects were evident in the lungs of hyperoxia exposed pups. There were no significant differences in IL-10 concentration in the lungs of hyperoxia-PL or hyperoxia male MSC treated groups, Fig 6a. In contrast, treatment of hyperoxia exposed pups with female MSC significantly increased lung IL-10 concentrations (66 ± 10 vs 59 ± 8 vs 80 ± 10, pg/mL, hyperoxia-PL vs hyperoxia male MSC vs hyperoxia female MSC; *P* < 0.05, N = 5-6/group), Fig 6a.

Furthermore, evaluation of IL-1β expression, a pro-inflammatory cytokine known to play an important role in BPD pathogenesis [34, 35], demonstrated that the protein expression of this cytokine was significantly increased in lung homogenates obtained from hyperoxia-PL animals (RA vs hyperoxia-PL; *P* < 0.05, N = 5/group). In contrast, while treatment with male or female MSC decreased lung IL-1β expression, this was more marked in hyperoxic pups which received female MSC (hyperoxia male MSC vs hyperoxia female MSC; *P* < 0.05, N = 4–6 / group), Fig 6b and 6c.

These differences in anti-inflammatory properties between male and female MSC were prominent in hyperoxia exposed male pups receiving female MSC. They had greater pulmonary IL-10 concentration than male MSC treated animals (56 ± 10 vs 83 ± 12 pg/mL; hyperoxia male MSC vs hyperoxia female MSC; *P* < 0.05, N = 4-5/group). Similar trends were noted in female pups receiving female MSC, but the differences were not statistically significant (61 ± 2 vs 78 ± 16 pg/mL; hyperoxia male MSC vs hyperoxia female MSC; N = 4/group).

This suggests that female MSC decreases lung inflammation in experimental BPD to a greater degree than male MSC.

### Female and Male MSC exhibit similar rates of engraftment

MSC from female human donors have been shown to be more clonogenic and to express more vascular cell adhesion molecule 1 than MSC obtained from male donors [22]. However, whether female MSC engraft more than male MSC in the lungs of neonatal pups with hyperoxia-induced lung injury is unknown. We assessed MSC engraftment using GFP^pos^ immunostaining. There was no difference in lung engraftment rates between male or female MSC in hyperoxic pups (0.72 ± 0.95 vs. 1.28 ± 1.81 GFP^pos^ cells per 1000 lung cells, hyperoxia male MSC vs hyperoxia female MSC; N = 10/group).

## Discussion

Over the last decade, cell-based therapies have been investigated as a potential strategy to decrease BPD. The most efficacious cellular population has yet to be elucidated. In the present study, we provide new evidence that female BM-derived MSC have greater therapeutic efficacy than male MSC in reducing neonatal hyperoxia-induced lung inflammation and vascular remodeling. Furthermore, the beneficial effects of female MSC were more pronounced in male animals. These findings have significant clinical implications as cell-based therapies for preterm infants with severe BPD/PH move from the bench to the bedside.

The hallmarks of BPD are alveolar simplification, vascular pruning and remodeling [36]. Despite strategies to limit lung injury, BPD still occurs in 30% of infants < 1,000g and results in significant morbidity and mortality, particularly if complicated by PH [37]. In a study on Medicaid health care expenditure for the care of children with chronic illnesses in Washington State, chronic respiratory diseases cost the program in excess of 17 million dollars, with the majority of these children having BPD or sequelae of prematurity [38]. Urgent therapies are therefore needed.

In rodent models, MSC are efficacious in reducing experimental BPD complicated by PH [18–20]. Furthermore, a dose escalation clinical trial in high-risk neonates demonstrated the short-term safety of intra-tracheal MSC delivery [21]. MSC possess anti-inflammatory, pro-angiogenic, anti-oxidant, and anti-fibrotic properties [19, 39]. However, given the heterogeneous nature of MSC, the most efficacious MSC subset for the treatment of BPD needs to be elucidated prior to their clinical use.

MSC possess sex hormone receptors [22, 40], and this has been suggested to influence their efficacy. Estradiol treated male MSC are more effective than untreated MSC in animal models of multiple sclerosis and cardiac injury [41, 42]. Moreover, in an endotoxin-induced myocardial injury model, Manukyan et al. demonstrated the superiority of female MSC in ameliorating injury [26]. These differences are potentially secondary to variation in signaling pathways that modulate cytokine production in male and female MSC. Indeed, Crisostomo et al. demonstrated that stress activated female MSC have lower TNF production as compared to male MSC, and these differences were due to an inherent resistance to TNFR1 activation in female MSC [27].

In our study, we also demonstrate that female MSC have more anti-inflammatory effects as compared to male MSC. We show that female MSC secrete more IL-10, and hyperoxia-exposed pups treated with female MSC had higher lung IL-10 concentration as compared to pups that received male MSC. This was accompanied by lower expression of the pro-inflammatory cytokine IL-1β in the lungs of hyperoxic pups that received female MSC. MSC are known to secrete IL-10 and several studies have demonstrated the important role of IL-10 in MSC-mediated organ repair. Burchfield et al showed that MSC secretion of IL-10 played a central role in promoting myocardial repair [43]. IL-10 is a pleiotropic regulatory cytokine which downregulates the expression of several pro-inflammatory cytokines, including IL-1β, and exogenous administration of recombinant IL-10 alleviated HILI in rodents [44].

Interestingly, the greater anti-inflammatory effect of female MSC conferred a marked benefit on the pulmonary vasculature. In our study, pups with hyperoxia-induced lung injury that received female MSC had a more robust attenuation in PH and degree of pulmonary vascular remodeling. Pulmonary inflammation is known to cause pulmonary vascular remodeling [45, 46]. Treatment with anti-inflammatory agents reduces PH and vascular remodeling in several lung injury models [47]. Moreover, female MSC are superior to male MSC in reducing monocrotaline-induced PH [48].

Another important finding in our study is the restoration of lung VEGF concentration to normoxic levels by female MSC. This is in keeping with prior findings of increased VEGF production in female MSC made by Crisostomo et al [27]. VEGF is a pluripotent growth factor that plays an integral role in lung development. Neonatal hyperoxia-exposure decreases VEGF concentration [49, 50], and administration of recombinant VEGF improves neonatal hyperoxia-induced lung injury [51].

Intriguingly, in our study, sub-group analysis investigating gender donor-recipient interactions revealed that males with HILI benefited more from female MSC administration as compared to male MSC. This was accompanied by higher levels of pulmonary IL-10 and restoration of VEGF in this group of pups. This suggests that female MSC exert their superior regenerative properties by providing a more anti-inflammatory and proangiogenic state in the injured lung. Interestingly, our study did not demonstrate any difference in the degree of hyperoxia-induced lung injury between male and female rats. The profound lung injury in our model may have eliminated these differences in the host lungs. Other studies have however shown that male animals undergo more pronounced injury and develop a more severe form of PH [10]. This is likely mediated by the differential expression of genes in response to injury between the two sexes; with males up-regulating pro-inflammatory pathways [52]. We postulate that male animals benefited more from female MSC as a consequence of the differential gene expression in response to lung injury between the two sexes [52], and the potential effect of estradiol (E2) secreted by female MSC on the male recipient [53]. E2 has been shown to increase the efficacy of male MSC in other models of injury [41, 42].

It is also important to discuss the limitations of our study. Hyperoxia induced lung injury models parallel a more severe form of BPD which in now infrequently seen. Hence, it would be imperative to evaluate the efficacy of MSC from different sexes in milder lung injury models. We also studied engraftment using GFP^pos^ localization. The GFP transgene is however unstable and as a result may have underestimated the degree of engraftment. Additionally, hyperoxia exposes multiple organs to injury through systemic inflammation and formation of circulating reactive oxygen species. It would therefore be crucial to examine the differential systemic effects of IT-MSC and any potential long-term side effects in future studies.

Our study however clearly demonstrates the superiority of female MSC in reducing lung inflammation, improving PH and hyperoxia-induced pulmonary vascular remodeling. These benefits were particularly evident in male animals with even more pronounced anti-inflammatory effects evidenced in these recipients. Our present findings have important implications as cell-based therapies to repair the injured preterm lung move from the bench to the bedside, providing firm evidence that female MSC are the more efficacious population to reduce severe BPD complicated by PH in male preterm infants.
